# Role of Bimetallic Solutions in the Growth and Functionality of Cu-BTC Metal–Organic Framework

**DOI:** 10.3390/ma15082804

**Published:** 2022-04-11

**Authors:** Nishesh Kumar Gupta, Jiyeol Bae, Kwang-Soo Kim

**Affiliations:** 1 Department of Environmental Research, University of Science and Technology (UST), Daejeon 34113, Korea; guptan@kict.re.kr (N.K.G.); baejiyeol@kict.re.kr (J.B.); 2 Department of Land, Water and Environment Research, Korea Institute of Civil Engineering and Building Technology (KICT), Goyang 10223, Korea

**Keywords:** bimetallic, copper, metal–organic frameworks, spectroscopy

## Abstract

Bimetallic solutions play a vital role in the growth and functionality of copper trimesate (Cu-BTC) metal–organic frameworks (MOFs). The effect of Ag^+^, Ca^2+^, Mn^2+^, Co^2+^, and Zn^2+^ on the growth of Cu-BTC was studied by fabricating M-Cu-BTC MOFs at room temperature using bimetallic M-Cu solutions. While Ag^+^ in the MOF had a rod-like morphology and surface properties, divalent cations deteriorated it. Moreover, unconventional Cu^+^ presence in the MOF formed a new building unit, which was confirmed in all the MOFs. Apart from Ag and Mn, no other MOF showed any presence of secondary cations in the structure. While Ag-Cu-BTC showed an improved H_2_S uptake capacity, other M-Cu-BTC MOFs had superior organic pollutant adsorption behavior. Thus, we have demonstrated that the physicochemical properties of Cu-BTC could be modified by growing it in bimetallic solutions.

## 1. Introduction

Cu-BTC (MOF-199 or HKUST-1) is among the most studied metal–organic frameworks (MOFs), in which dimeric Cu^2+^ units are bridged by benzene-1,3,5-tricarboxylate (BTC) linkers. Cu-BTC has found numerous applications in CO_2_ capture [1], air purification [2], photocatalysis [3], and catalysis [4]. Nowadays, researchers have started exploring bimetallic MOFs with a second metal ion in Cu-BTC, which have been found to be superior to Cu-BTC in the desired applications [5,6,7,8]. Many of the studies have reported ion exchange of a fraction of Cu ions with the secondary metal ions post-Cu-BTC fabrication for the development of M/Cu-BTC MOFs [7,9]. In addition, solvothermal synthesis with binary metal solutions has been reported at elevated temperatures [10,11,12]. Among numerous methods adopted for the fabrication of Cu-BTC, room-temperature synthesis is known for being an industrially relevant protocol [13,14]. To the best of our understanding, room-temperature synthesis of M/Cu-BTC MOFs, which we have aimed to investigate in this study, is largely unexplored in the literature.

In the literature, Cu-BTC has been used for the adsorptive capture of toxic hydrogen sulfide (H_2_S) gas [2,15,16]. In our previously reported work, we have demonstrated that the inclusion of silver (Ag^0^ and Ag^+^) in the Cu-BTC structure significantly improved its H_2_S uptake capacity and stability against this corrosive gas [17]. Thus, it could be novel to correlate the physicochemical changes that occurred in Cu-BTC (due to bimetallic solutions) with its H_2_S adsorption capacity. Similarly, this MOF has been widely studied for the adsorptive removal of organic molecules, such as methylene blue (MB) dye [18,19] and pharmaceutical drugs [20,21]. The adsorption of these bulkier organic molecules is highly dependent on the surface area and pore characteristics of the MOF. Since the inclusion of secondary cations in the MOF influences the surface and pore characteristics, organic molecules of environmental concern (dyes and drugs) could be effective probe molecules to address these characteristics. Moreover, this would ensure that such modifications are suitable for improving the MOF behavior towards adsorbing toxic organic molecules.

In this study, we have reported the role of bimetallic solutions in the growth and functionality of M-Cu-BTC at room temperature. These MOFs were synthesized by the simple mixing of organic linker solution in bimetallic solution in ambient conditions, with a much smaller amount of organic solvent, to make the adsorption process affordable and environmentally benign. The synthesized MOFs were well characterized by various microscopic and spectroscopic techniques, to understand the differences in their physicochemical properties. These MOFs were studied for the adsorptive removal of H_2_S gas, MB dye and diclofenac sodium (DCF) in the gaseous and aqueous phases, respectively. The study showed a large variation in the uptake capacities of these MOFs, which was largely driven by the surface area and pore characteristics of the MOFs. Thus, we successfully demonstrated that the physicochemical properties and adsorption behavior of Cu-BTC MOF could easily be altered by fabricating it in bimetallic solutions.

## 2. Materials and Methods

### 2.1. Chemicals

Copper(II) nitrate trihydrate (Cu(NO_3_)_2_·3H_2_O), silver(I) nitrate (AgNO_3_), calcium(II) nitrate tetrahydrate (Ca(NO_3_)_2_·4H_2_O), cobalt(II) nitrate hexahydrate (Co(NO_3_)_2_·6H_2_O), manganese(II) nitrate tetrahydrate (Mn(NO_3_)_2_·4H_2_O), zinc(II) nitrate hexahydrate (Zn(NO_3_)_2_·6H_2_O), benzene-1,3,5-tricarboxylic acid (H_3_BTC), and methylene blue (MB) were purchased from Sigma Aldrich, Taufkirchen, Germany. N,N-dimethyl formamide (DMF) was procured from Samchun Pure Chemicals, Pyeongtaek-si Korea. Diclofenac sodium salt (DCF, C_14_H_10_Cl_2_NNaO_2_) was procured from TCI Chemicals, Tokyo, Japan. Pure H_2_S (500 ppm) balanced with N_2_ gas was procured from Union gas, Seoul, Korea.

### 2.2. Synthesis of MOFs

Cu-BTC was synthesized at room temperature by adding a H_3_BTC solution (4.2 g in 0.2 L solution of DMF: H_2_O ~1:3 (*v*/*v*)) in Cu salt solution (7.248 g in 0.2 L of distilled water) and stirring for 24 h. The precipitate was separated by centrifugation, washed twice with distilled water, and dried at 70 °C for 48 h. The effect of secondary metals on the growth of Cu-MOF was studied by synthesizing MOFs using the same protocol with bimetallic solutions. For Ag-Cu-BTC, a binary salt solution, containing 7.0 g of Cu salt and 0.17 g of Ag salt, was used. A binary salt solution containing 7.0 g of Cu salt along with 0.236 g of Ca, 0.251 g of Mn, 0.291 g of Co, and 0.298 g of Zn salt was used for Ca-Cu-BTC, Mn-Cu-BTC, Co-Cu-BTC, and Zn-Cu-BTC, respectively.

### 2.3. Analytical Instruments

The morphology of MOFs was analyzed by field emission scanning electron microscopy (FE-SEM, Hitachi S-4300, Hitachi, Tokyo, Japan) and field emission transmission electron microscopy (FE-TEM, JEM-2010F, JEOL Ltd., Tokyo, Japan). Elemental mapping was performed using energy-dispersive X-ray spectroscopy (EDS) (X-Maxn 80T, Oxford Instruments, Abingdon, UK) in TEM mode. The powder X-ray diffraction patterns were obtained at room temperature (2θ = 5–50°) on an Ultima IV (Rigaku, Tokyo, Japan) X-ray diffractometer with Cu Kα radiation (λ = 1.5406 Å) and a Ni filter, where the scanning speed was set to 3° min^−1^. Thermal gravimetric analysis (TGA) was performed using a Thermogravimetric Analyzer (TG 209 F3, NETZSCH-Gerätebau GmbH, Waldkraiburg, Germany). The specific surface area and porosity of MOFs were determined by analyzing the standard N_2_ adsorption–desorption isotherm at −196 °C using a Gemini 2360 series (Micromeritics, Norcross, GA, USA) instrument after degassing at 200 °C for 6 h. Fourier transform infrared (FT-IR) spectra were recorded using an Cary670 FTIR spectrometer (Agilent Technologies, Santa Clara, CA, USA) in the frequency range of 400–4000 cm^−1^. X-ray photoelectron spectroscopy (XPS: K-alpha XPS instrument, Thermo Fisher Scientific, East Grinstead, UK) was employed to determine the elemental compositions and chemical states of the prepared MOFs. For XPS analysis, a monochromatic Al Kα X-ray source was used with a fixed pressure of 4.8 × 10^−9^ mbar. Spectra were charge corrected to the main line of the C 1s spectrum (aromatic carbon) set to 284.7 eV. Spectra were analyzed using CasaXPS software (version 2.3.14) with GL(*p*) = Gaussian/Lorentzian product formula, where the mixing is determined by *m* = *p*/100, GL(100) is a pure Lorentzian, while GL(0) is a pure Gaussian. We used GL(30).

### 2.4. Experimental Methodology

Gas breakthrough studies: The adsorption experiments were performed by taking 250 mg of a powdered MOF in a Pyrex tube (height: 50 cm and diameter: 1 cm) at 25 °C. The MOF sample was placed between the glass wool and supported on silica beads. The H_2_S gas (500 ppm) was passed through it at a flow rate of 0.2 L min^−1^. The outgoing gas was analyzed using a gas analyzer (GSR-310, Sensoronic, Bucheon, Korea). The H_2_S concentration was measured every 15 s until the effluent concentration reached 10 ppm (Figure S1). The gas adsorption capacity was measured using the following equation [22]:

$$q=\frac{C_{0}Q}{m}\int_{0}^{t_{b}}(1-\frac{C}{C_{0}})dt$$

where *C*_0_—initial concentration (mg L^−1^), *C*—concentration at a time ‘*t*’ (mg L^−1^), *Q*—flowrate, *m*—the mass of MOF (g), and *t*_b_—breakthrough time.

MB/DCF adsorption experiments: The adsorption experiments were performed at room temperature under sonication. Exactly 60 mg of an MOF was placed in a 50 mL plastic vial and sonicated with 30 mL of MB dye or DCF solution (50 mg L^−1^) at a neutral pH. Aliquots of the aqueous phase were taken out at a fixed time and analyzed by UV–Vis spectroscopy (LAMBDA 365 UV/Vis Spectrophotometer, Perkin Elmer, Waltham, MA, USA) after suitable dilution. The pollutant concentration in the aqueous phase was estimated from the peak at λ = 664 and 276 nm for MB dye and DCF, respectively. The adsorption capacity at any time ‘*t*’ (*q*_t_, mg g^−^^1^) was calculated using the following equation:

$$q_{t}=\frac{\left(C_{0}-C_{t}\right)*V}{m}$$

where *C*_0_—initial concentration (mg L^−1^), *C*_t_—concentration at a time ‘*t*’ (mg L^−1^), *V*—volume (mL), and *m*—the mass of MOF (g).

## 3. Results and Discussion

**Figure 1:**
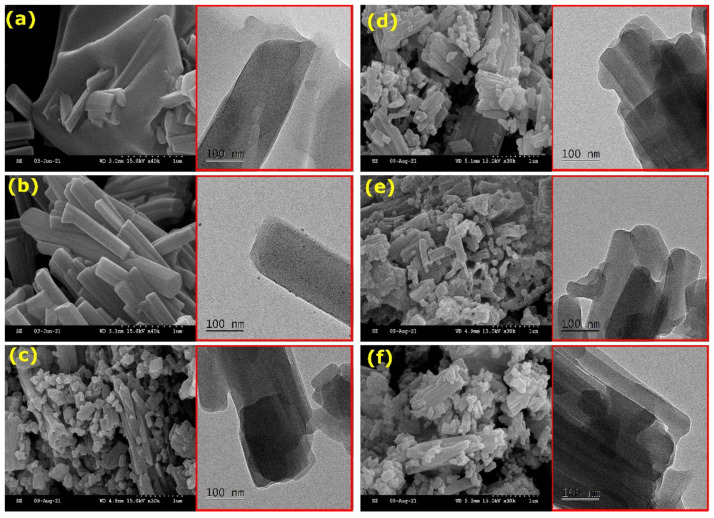
SEM and HR-TEM images (in red box) of (**a**) Cu-BTC; (**b**) Ag-Cu-BTC; (**c**) Ca-Cu-BTC; (**d**) Mn-Cu-BTC; (**e**) Co-Cu-BTC; (**f**) Zn-Cu-BTC.

The M-Cu-BTC (M: Ag^+^, Ca^2+^, Mn^2+^, Co^2+^, and Zn^2+^) MOFs were synthesized by stirring bimetallic solutions (Cu^2+^: M^n+^ ~30) with the trimesic acid linker in the H_2_O:DMF solution for 24 h. The scanning electron microscopy (SEM) and transmission electron microscopy (TEM) images of Cu-BTC confirmed the formation of a rod-like morphology (Figure 1a) [2]. The effect of the bimetallic solution was visible on the morphology. The Ag-Cu solution led to the formation of smooth and uniform microrods (Figure 1b); whereas, the other binary solutions formed rough and non-uniform microrods (Figure 1c–f).

**Figure 2:**
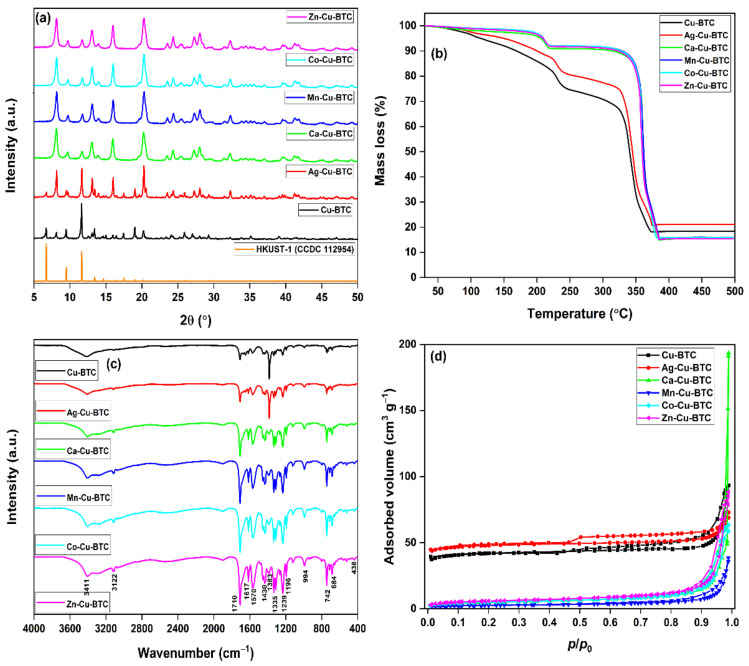
(**a**) PXRD patterns; (**b**) TGA profiles; (**c**) FTIR spectra; (**d**) N_2_ adsorption–desorption curves of MOFs.

The powder X-ray diffraction pattern (PXRD) of Cu-BTC matched well with the reported pattern of HKUST-1 (CCDC 112954) [23]. An additional peak, observed at 2θ = 8.1°, hinted towards the formation of a new and independent building unit for Cu^I^ sites, probably as Cu^I^_2_(H_2_BTC)_2_. It is analogous to the behavior observed for Ag^I^ in [{Ag(H_2_BTC)_2_}{Ag_2_(HBTC)}]_n_, constructed of Ag chains formed by ligand unsupported Ag–Ag interactions and H_2_BTC^−^ and HBTC^2−^ spacers [24]. The peak intensity at 8.1° increased in Ag-Cu-BTC, with a new peak at 9.8°. These changes were associated with the independent, yet identical (to the Cu^I^ sites), building unit for Ag^I^ sites in the MOF [17]. In other MOFs, the peak at 6.7° disappeared and the intensity of the peak at 8.1° improved (compared to Cu-BTC), which was due to a higher proportion of Cu^I^ sites in the MOF. Though in Ca-Cu, Co-Cu, and Zn-Cu MOFs, the secondary metal was not traced by XPS analysis, these secondary cations during the synthesis phase significantly altered the MOF structural units (Figure 2a). However, the presence of Mn ions formed Mn-Cu-BTC with a PXRD pattern similar to the reported pattern for Mn(II)-impregnated HKUST-1 [25]. The thermogravimetric analysis (TGA) profiles of Cu-BTC and Ag-Cu-BTC have three mass loss stages, for the evaporation of water (30–220 °C), the removal of solvent molecules (220–320 °C), and the breakdown of metal–linker coordination (320–380 °C). The TGA profiles of the other four MOFs showed only ~8% mass loss until 320 °C, which indicated a low presence of solvent/water molecules (Figure 2b) [2].

The Fourier transform infrared (FTIR) spectra have a broad intense band at 3411 cm^−1^ for the *v*_s_(OH) mode of H_2_O molecules. The asymmetric (1570 and 1617 cm^−1^) and symmetric (1383 and 1430 cm^−1^) bands were assigned to –C=O (BTC linker). The 1710 cm^−1^ band was attributed to the free –COOH groups (as confirmed by the FTIR spectrum of H_3_BTC in Figure S2) [26]. The intensity of this band increased for M-Cu-BTC (compared to Cu-BTC), which further confirmed the involvement of H_2_BTC^–^ and HBTC^2−^ spacers in the MOF framework. Multiple bands in the 1200–700 cm^−1^ region, observed for C–H bending modes, were coupled with the 3122 cm^−1^ band for the stretching C–H vibrations (Figure 2c) [27]. The isotherms and pore size distribution were analyzed by a N_2_ adsorption–desorption isotherm at −196 °C (Figure 2d and Figure S3). Cu-BTC and Ag-Cu-BTC exhibited a type IVa isotherm for mesoporous adsorbents. On the contrary, isotherms for other M-Cu-BTC MOFs were identified for mesoporous or macroporous materials [19,28]. The surface area of 163 m^2^ g^−1^ (Cu-BTC) improved for Ag-Cu-BTC (188 m^2^ g^−1^), and then remained in the range of 9–21 m^2^ g^−1^ for the other MOFs (Table 1). The average pore size (*D*_p_) in Cu-BTC (3.5 nm) and Ag-Cu-BTC (2.4 nm) confirmed the mesoporosity in the MOFs. For Ca-Cu-BTC, *D*_p_ (~70.1 nm) was in the macroporous range (>50 nm); while, in the other samples, the *D*_p_ was in the range of 22.0–26.1 nm (mesoporosity) [28]. Thus, the secondary cation (except for Ag) in the binary solutions destroyed the mesoporosity of Cu-BTC.

**Figure 3:**
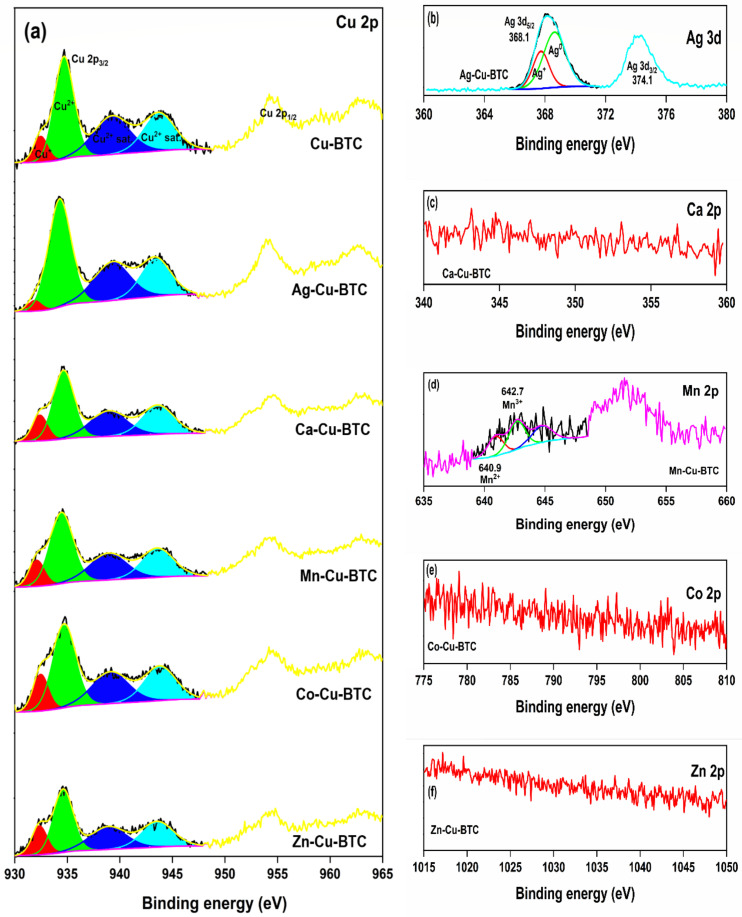
HRXPS (**a**) Cu 2p spectra of MOFs; (**b**) Ag 3d spectrum in Ag-Cu-BTC; (**c**) Ca 2p spectrum in Ca-Cu-BTC; (**d**) Mn 2p spectrum in Mn-Cu-BTC; (**e**) Co 2p spectrum in Co-Cu-BTC; (**f**) Zn 2p spectrum in Zn-Cu-BTC.

The X-ray photoelectron spectroscopy (XPS) analysis confirmed that, apart from Ag (1.4%) in Ag-Cu-BTC and Mn (0.8%) in Mn-Cu-BTC, no other M-Cu-BTC sample showed the presence of respective secondary cations (Figure S4 and Table S1). The same inference was confirmed by TEM-EDAX analysis, where Ca-Cu-BTC, Co-Cu-BTC, and Zn-Cu-BTC showed no presence of secondary metal ions (Figure S5). Cu might have had stronger coordination with the carboxylate groups in these MOFs, which would have limited the coordination of the secondary cation during the MOF synthesis process. The high-resolution XPS (HRXPS) Cu 2p spectra of MOFs are shown in Figure 3a. For all the MOFs, the Cu 2p_3/2_ peak was deconvoluted into two contributions at 931.9–932.5 and 934.3–934.7 eV for the Cu^+^ and Cu^2+^ sites, respectively, with two satellite peaks for Cu^2+^ ions (Table S2) [15]. A comparatively higher Cu^+^ proportion in M-Cu-BTC (20.1–27.0%), over Cu-BTC (15.7%) (except Ag-Cu-BTC), was the reason for the increased PXRD peak intensity (2θ = 8.1° for a new Cu^I^–Cu^I^ building unit in the MOF). The %Cu atomic composition in these MOFs was nearly identical, which further confirmed this proposition. For Ag-Cu-BTC, though the Cu^+^ proportion was 5.4%, the total atomic Cu proportion was 2.4%. Moreover, the MOF has a fraction of Ag as Ag^+^, which led to comparatively higher intensity for the 8.1° peak. The HRXPS Ag 3d spectrum in Ag-Cu-MOF has two peaks at 368.1 and 374.1 eV for 3d_5/2_ and 3d_3/2_, respectively, with spin–orbit coupling of 6.0 eV (Figure 3b). The 3d_5/2_ peak was deconvoluted into two contributions at 367.7 and 368.6 eV for Ag^+^ (32.5%) and Ag^0^ (67.5%), respectively [29]. The HRXPS Ca 2p (Figure 3c), Co 2p (Figure 3e), and Zn 2p (Figure 3f), in their respective M-Cu-BTC MOFs, confirmed no signals. The presence of Mn in Mn-Cu-BTC was confirmed by low-intensity signals for Mn 2p (Figure 3d) and Mn 3s (Figure S6). The HRXPS Mn 2p spectrum of Mn-Cu-BTC has two contributions at 640.9 and 642.7 eV for Mn^2+^ (40%) and Mn^3+^ (60%), respectively (Figure 3d) [30].

**Figure 4:**
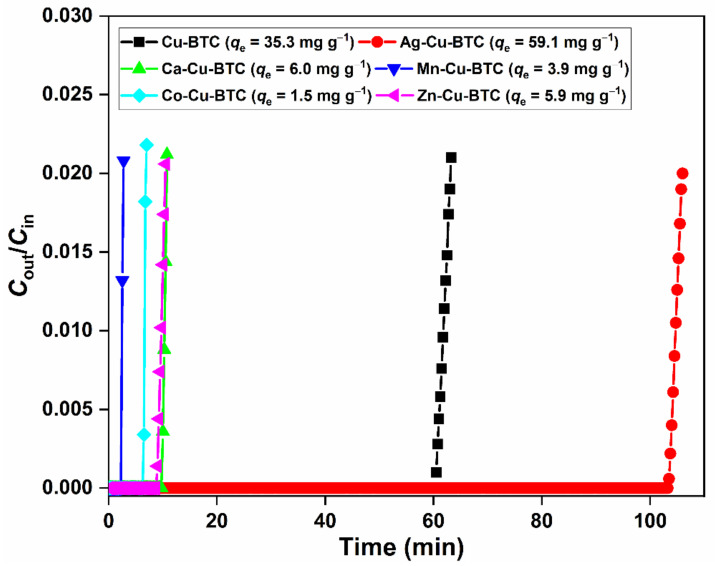
H_2_S breakthrough curves of MOFs. [H_2_S] = 500 ppm, flow rate = 0.2 L min^−1^, [MOF] = 0.25 g, and T = 25 °C.

The effect of bimetallic solutions on the adsorption properties of fabricated MOFs was studied, with H_2_S as a gaseous pollutant (Figure 4). Cu-BTC has been studied for H_2_S removal on numerous occasions, where the H_2_S molecules show reactive binding with the open Cu sites (Table S3) [15,16]. Thus, H_2_S is an ideal pollutant to understand the changes arising from bimetallic solutions. The H_2_S adsorption capacity of Cu-BTC (35.3 mg g^−1^) improved in Ag-Cu-BTC (59.1 mg g^−1^). For the other MOFs, this value was in the 1.5–6.0 mg g^−1^ range. This behavior matched well with the trend observed for surface area, where Ag-Cu-BTC, with the maximum surface area, had the highest H_2_S adsorption capacity (Table 1). In addition, the total metal content in Ag-Cu-BTC was significantly higher than other MOFs, which played a dominant role in the H_2_S adsorption process (Table S1). Since H_2_S is a small molecule (kinetic diameter ~3.6 Å) [31], diffusion occurred irrespective of the pore size.

**Figure 5:**
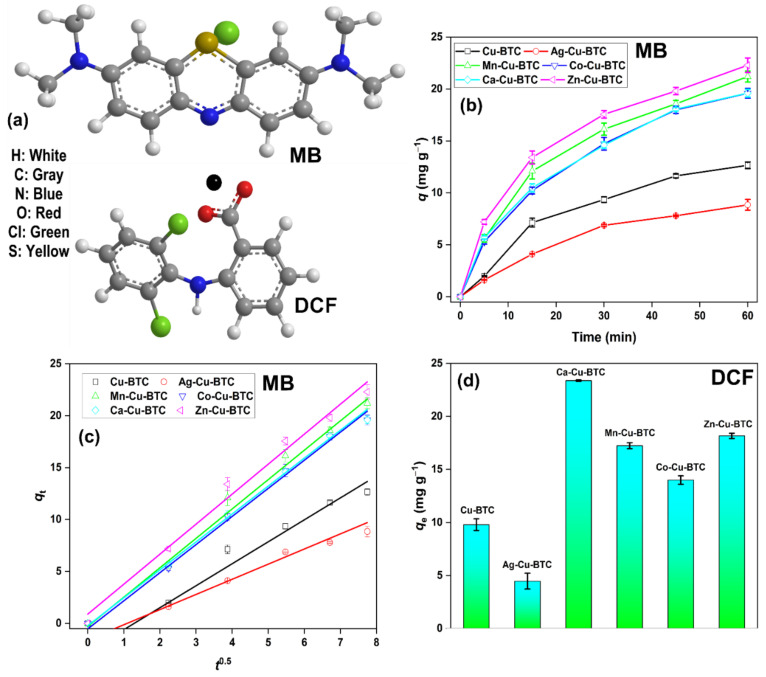
(**a**) Molecular structure of MB and DCF; (**b**) MB dye adsorption behavior of synthesized MOFs; (**c**) kinetic data fitted to the IPD model; (**d**) DCF adsorption capacity of MOFs after 1 h of sonication.

The role of pore size is vital for the adsorption of MB dye (length ~14.5 Å; width ~9.5 Å) [18] and DCF molecules (length ~9.1 Å; width ~7.0 Å) [32], which are bigger than H_2_S molecules [18]. MB dye (cationic dye) is adsorbed via a cation-exchange mechanism with the metal ions in HKUST-1 [9]. In addition, π-π interactions between the aromatic rings of the MB dye and the organic linker in the MOF are responsible for the adsorption process [33]. DCF adsorption over these MOFs was studied to diversify the applicability of these MOFs for the removal of emerging pharmaceutical pollutants. DCF is an anionic molecule at a neutral pH, and is adsorbed via electrostatic interactions (cation-π) and π-π interactions (Figure 5a) [32]. The MB adsorption capacity of 12.7 mg g^−1^ (for Cu-BTC) dipped to 8.9 mg g^−1^ for Ag-Cu-BTC, due to decreased pore size and slow MB diffusion through these narrow pores. On the contrary, the other MOFs showed a comparatively higher adsorption capacity (19.6–22.3 mg g^−1^), due to the presence of larger-sized pores (Figure 5b) [9,18]. The kinetic data were fitted to the intra-particle diffusion (IPD) model to understand the rate of diffusion of dye molecules in the MOFs studied (Figure 5c) [34].

$$q_{t}=k_{ip}*\sqrt{t}+C_{i}$$

where *q_t_*—adsorption capacity at time ‘*t*’ (mg g^−1^), *k_ip_*—rate parameter (mg g^−1^ min^−1/2^), and *C_i_*—intercept that gives an idea about the thickness of the boundary layer (mg g^−1^).

The derived values for the kinetic parameters are listed in Table S4. Since the adsorption process was largely driven by the diffusion of dye molecules in the MOF structure, the experimental data fitted well with the IPD model, with a linear regression value in the range of 0.99–1.00. Moreover, the non-zero value for *C*_i_ suggested that IPD was not the only rate-limiting step. The lowest *k*_ip_ value of 1.46 mg g^−1^ min^−1/2^ was observed for Ag-Cu-BTC, which was due to it having the lowest pore diameter. Thus, even with the highest surface area, Ag-Cu-BTC showed the lowest rate of diffusion and a low MB adsorption capacity. On the contrary, other M-Cu-BTC MOFs possessed a *k*_ip_ value between 2.69 and 2.89 mg g^−1^ min^−1/2^.

**Figure 6:**
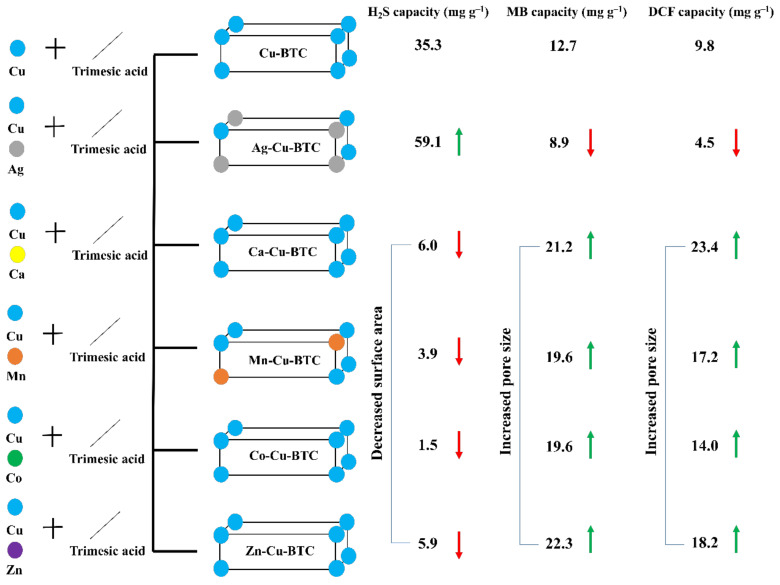
Summary of the present work.

A higher rate of MB diffusion in these MOFs could be supported by a larger pore diameter. The slight variation in the adsorption capacity trend among these MOFs could be due to the surface adsorption of MB molecules via π-π interactions, which is governed by the surface area. This inference is largely derived from the fact that these MOFs possessed a higher *C_i_* value, which represented a larger contribution of the surface adsorption in the rate-limiting step [35]. The results demonstrated the importance of pore size, and not surface area, in the MB diffusion process. Similarly, DCF adsorption showed a similar trend in the adsorption capacity after 1 h of sonication (Figure 5d). The DCF adsorption capacity in Cu-BTC (9.8 mg g^−1^) dropped to 4.5 mg g^−1^ in Ag-Cu-BTC. Other M-Cu-BTC MOFs showed an adsorption capacity between 14.0 and 23.4 mg g^−1^, which is in accordance with the trend observed for MB dye. The maximum adsorption capacity of 23.4 mg g^−1^ was reported for Ca-Cu-BTC, with the highest pore size of ~70 nm. Thus, it is possible to alter the physiochemical properties of Cu-BTC simply by using bimetallic solutions during the MOF fabrication process. Moreover, these modifications could help improve the MOF behavior in adsorbing existing and emerging organic pollutants. Additionally, it is possible to improve the H_2_S gas capture efficiency of Cu-BTC MOF by incorporating a small, yet significant, proportion of Ag. The outcome of the study has been simplified pictorially in Figure 6.

## 4. Conclusions

In this study, we have demonstrated the role of bimetallic solutions in the physiochemical characteristics and adsorption behavior of Cu-BTC. M-Cu-BTC MOFs were fabricated using M-Cu (M: Ag^+^, Ca^2+^, Mn^2+^, Co^2+^, and Zn^2+^) bimetallic solutions at room temperature. Ag-Cu-BTC showed a smooth rod-like morphology, which deteriorated in other bimetallic solutions. The PXRD analyses of MOFs showed a new and secondary Cu^I^–Cu^I^ building unit in the MOF framework. While Ag in the MOF improved the surface and pore characteristics, divalent secondary cations lowered the surface area and increased the pore diameter. Based on the XPS analysis, only Ag-Cu-BTC and Mn-Cu-BTC demonstrated the presence of secondary cations in the MOF framework. These alterations in the properties were tracked and quantified by adsorbing H_2_S gas, MB dye, and DCF pollutant. While the H_2_S adsorption capacity was higher for Ag, a low capacity was reported for the other M-Cu-MOFs. An inverse trend was confirmed for MB and DCF adsorption, where Ag-Cu-BTC adsorbed the least. Thus, this study conclusively presented the idea of modifying Cu-BTC properties by adopting bimetallic solutions during the MOF fabrication process.

## Figures and Tables

**Table 1:** Surface area and pore characteristics of MOFs.

MOF	*S*_BET_ (m^2^ g^–1^)	*V*_p_ (cm^3^ g^–1^)	*D*_p_ (nm)
Cu-BTC	163.43	0.144	3.5
Ag-Cu-BTC	187.64	0.113	2.4
Ca-Cu-BTC	17.08	0.299	70.1
Mn-Cu-BTC	9.23	0.060	25.9
Co-Cu-BTC	17.79	0.098	22.0
Zn-Cu-BTC	20.91	0.136	26.1

## Data Availability

Not applicable.

## Supplementary Materials

The following supporting information can be downloaded at: https://www.mdpi.com/article/10.3390/ma15082804/s1, Figure S1: Schematic representation of H_2_S adsorption system used in the study. Figure S2: FTIR spectrum of H_3_BTC ligand. Figure S3: Pore size distribution curves of M-Cu-BTC MOFs. Figure S4: XPS surveys of M-Cu-BTC MOFs. Figure S5: TEM-EDAX analysis of Ca-Cu-BTC, Co-Cu-BTC, and Zn-Cu-BTC. Figure S6: HRXPS spectrum of Mn 3s in Mn-Cu-BTC. Table S1: Surface elemental analysis using XPS analysis. Table S2: HRXPS Cu 2p signals of MOFs. Table S3: Room temperature H_2_S adsorption capacity comparison with the reported Cu-BTC. Table S4: Kinetic parameters for the IPD model. References [2,15,16,17,36,37,38,39,40,41] are cited in the Supplementary Materials.
