# Supplementary material for: Role of Bimetallic Solutions in the Growth and Functionality of Cu-BTC Metal–Organic Framework
Source: Materials (Basel). 2022 Apr 11;15(8):2804. doi: 10.3390/ma15082804 (PMC9033043; doi:10.3390/ma15082804)
Supplement: Supplementary file 1 [file materials-15-02804-s001.zip › materials-1664220-supplementary.pdf]

materials

Supplementary material

Role of bimetallic solutions in the growth and functionality of Cu-BTC metal-organic framework

Nishesh Kumar Gupta[a,b], Jiyeol Bae[a,b] and Kwang Soo Kim[a,b*]

1. University of Science and Technology (UST), Daejeon, Republic of Korea
2. Department of Land, Water, and Environment Research, Korea Institute of Civil Engineering and Building Technology (KICT), Goyang, Republic of Korea

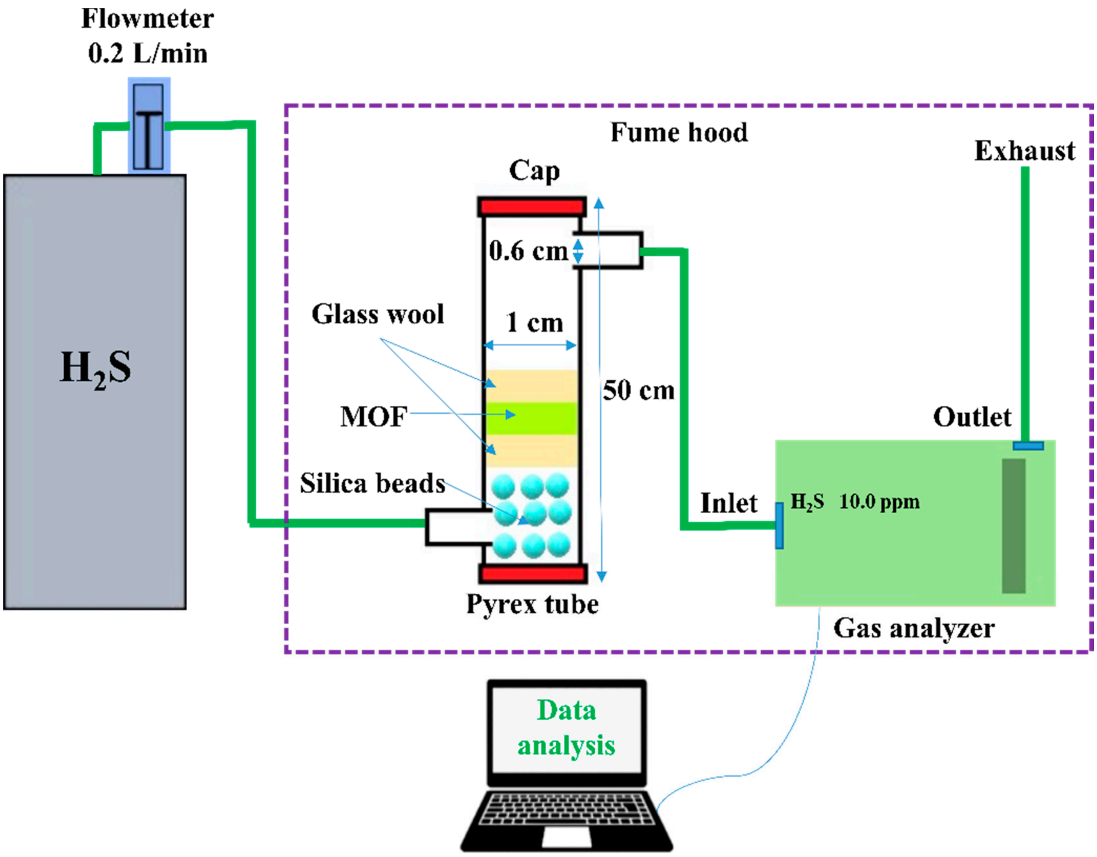

Figure S1. Schematic representation of H_2S adsorption system used in the study.

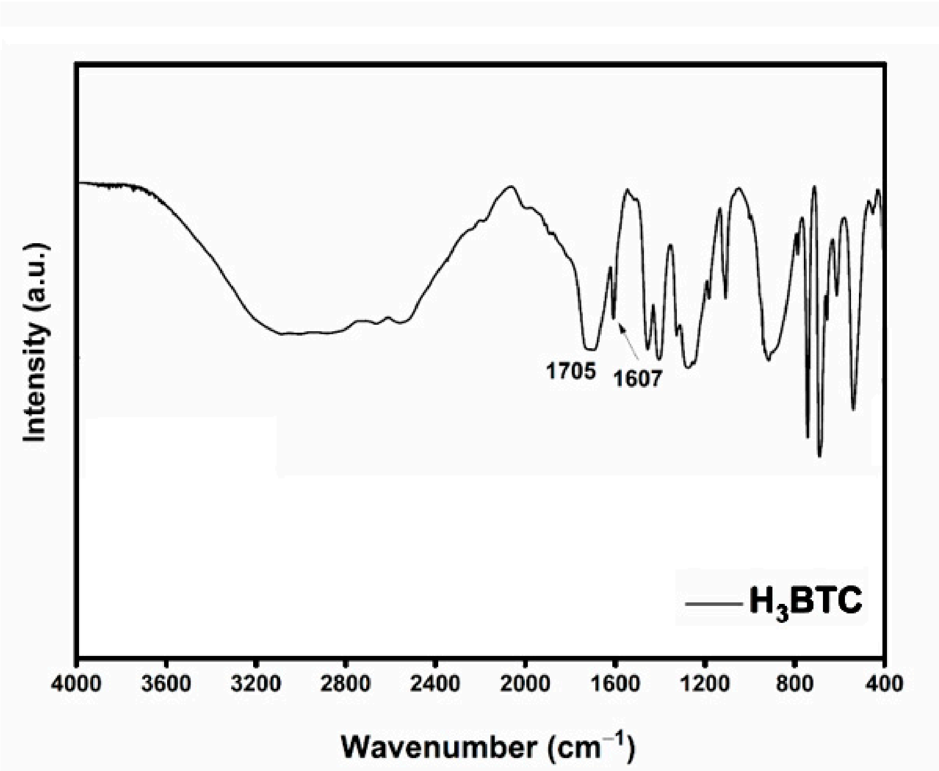

Figure S2. FTIR spectrum of H_3BTC ligand.

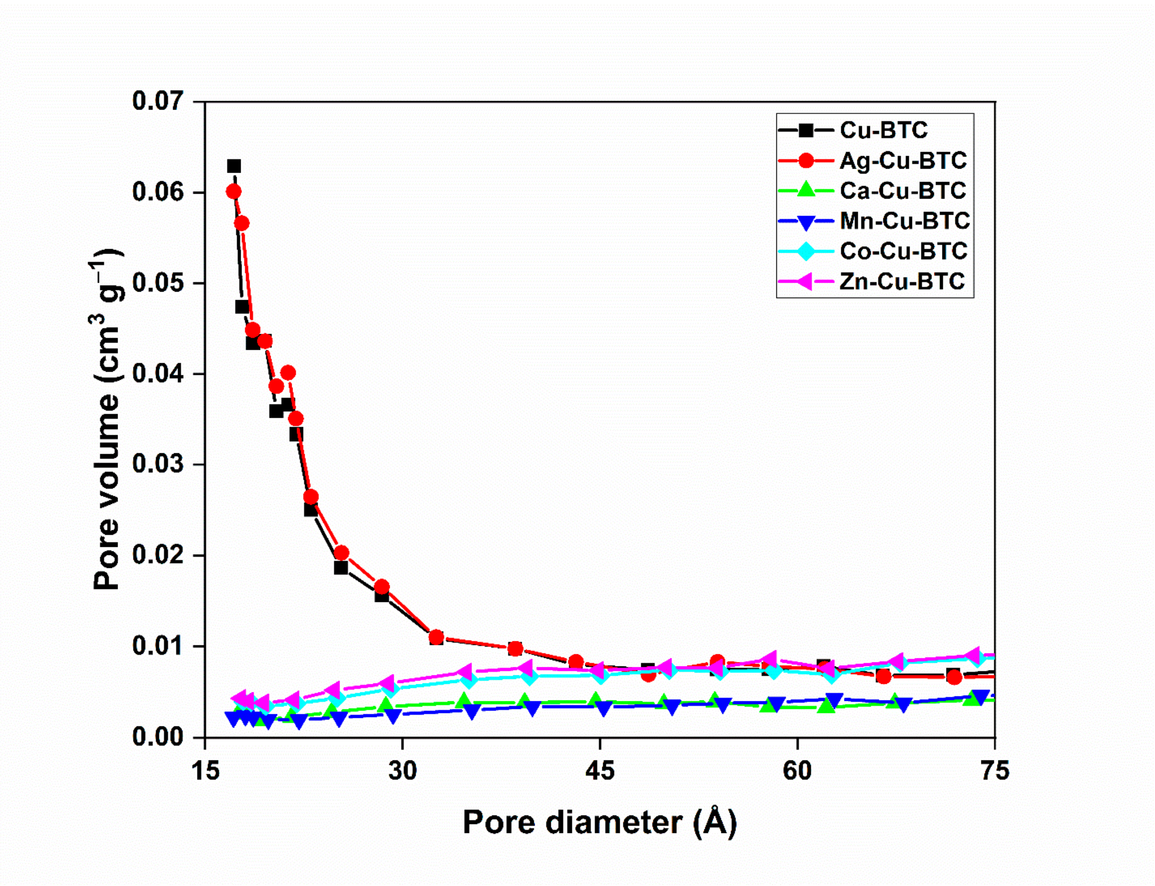

Figure S3. Pore size distribution curves of M-Cu-BTC MOFs.

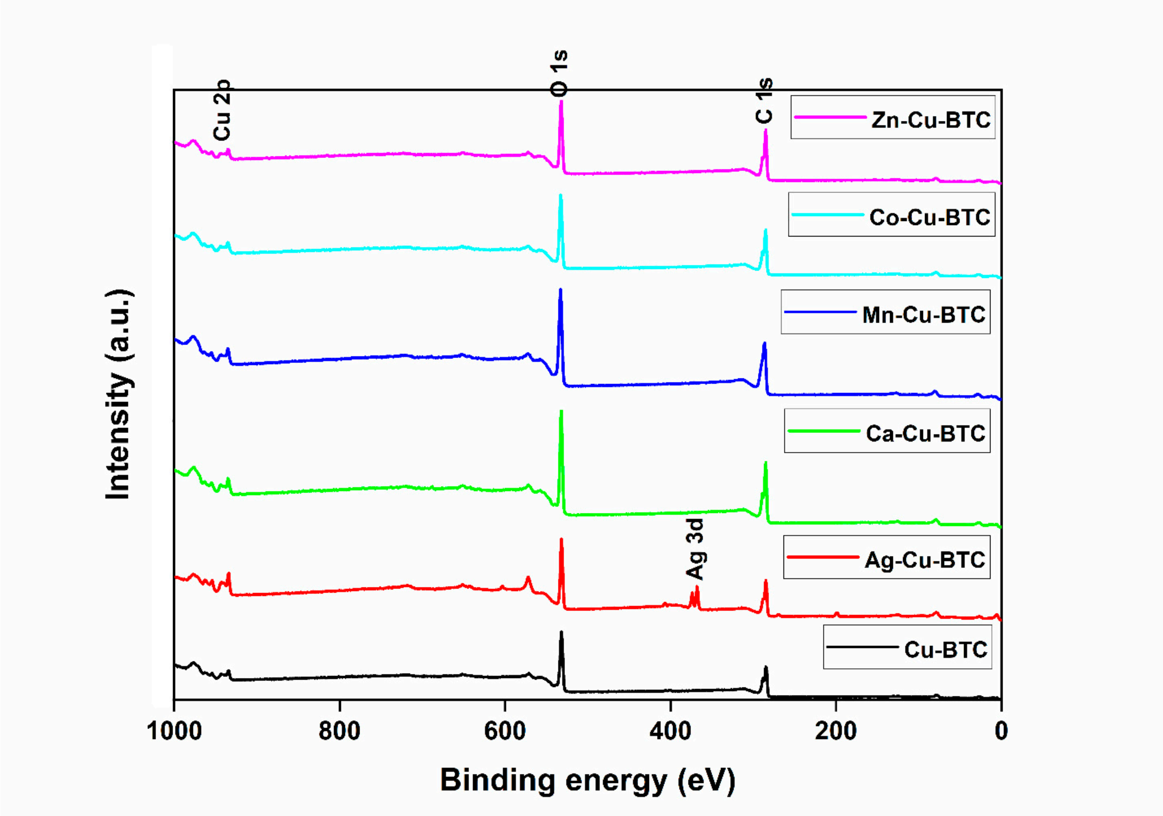

Figure S4. XPS surveys of M-Cu-BTC MOFs.

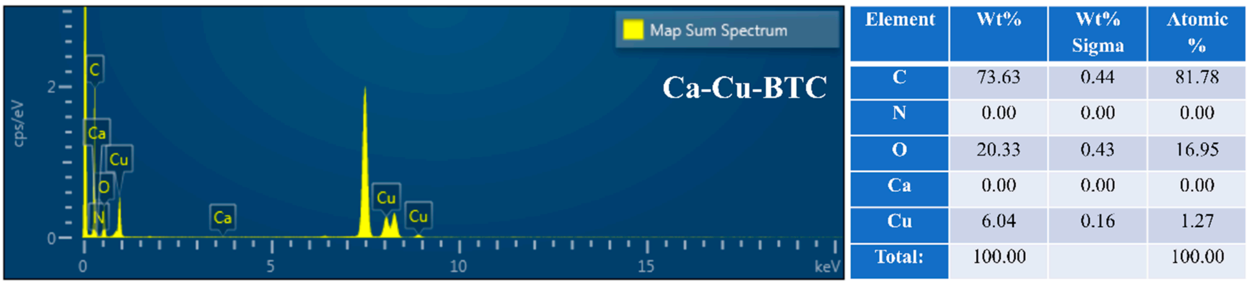

Element	Wt%	Wt% Sigma	Atomic %
C	73.63	0.44	81.78
N	0.00	0.00	0.00
O	20.33	0.43	16.95
Ca	0.00	0.00	0.00
Cu	6.04	0.16	1.27
Total:	100.00		100.00

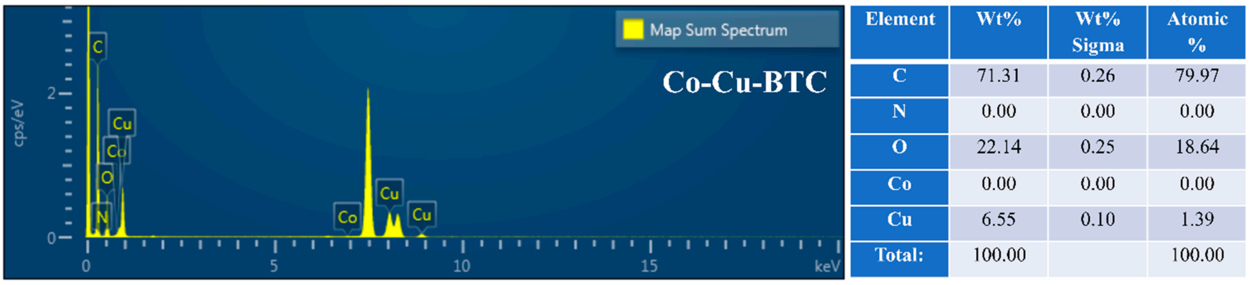

Element	Wt%	Wt% Sigma	Atomic %
C	71.31	0.26	79.97
N	0.00	0.00	0.00
O	22.14	0.25	18.64
Co	0.00	0.00	0.00
Cu	6.55	0.10	1.39
Total:	100.00		100.00

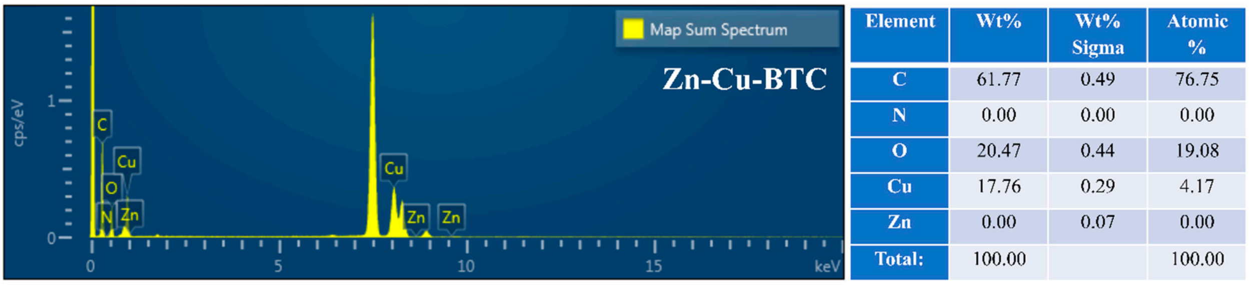

Element	Wt%	Wt% Sigma	Atomic %
C	61.77	0.49	76.75
N	0.00	0.00	0.00
O	20.47	0.44	19.08
Cu	17.76	0.29	4.17
Zn	0.00	0.07	0.00
Total:	100.00		100.00

Figure S5. TEM-EDAX analysis of Ca-Cu-BTC, Co-Cu-BTC, and Zn-Cu-BTC.

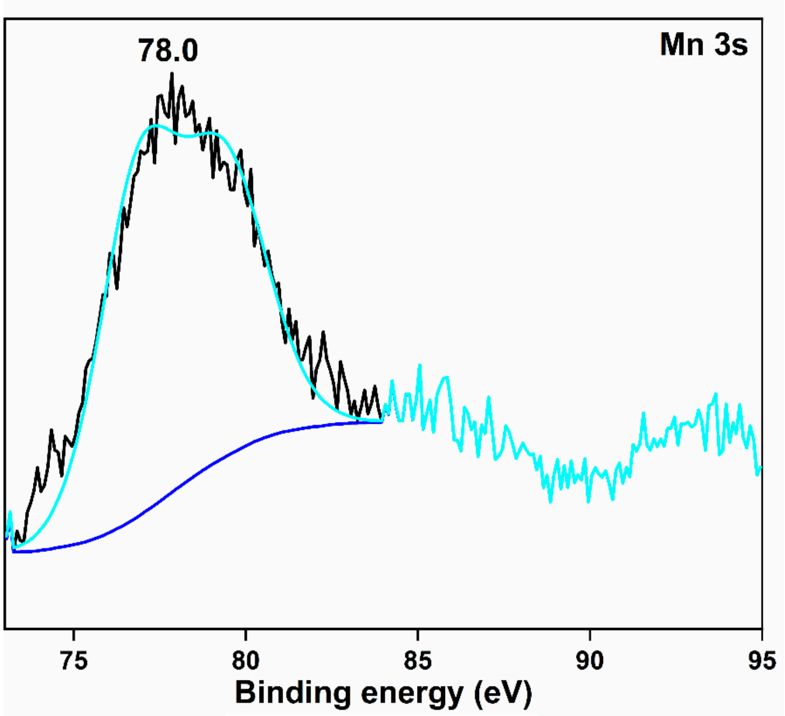

Figure S6. HRXPS spectrum of Mn 3s in Mn-Cu-BTC.

Table S1. Surface elemental analysis using XPS analysis.

MOF	Elements			
	C	O	Cu	Secondary Metal
Cu-BTC	64.8	33.7	1.5	-
Ag-Cu-BTC	65.0	31.2	2.4	1.4
Ca-Cu-BTC	65.8	32.7	1.5	No signal
Mn-Cu-BTC	65.6	32.2	1.4	0.8
Co-Cu-BTC	65.9	32.6	1.5	No signal
Zn-Cu-BTC	68.0	30.5	1.5	No signal

Table S2. HRXPS Cu 2p signals of MOFs.

Samples	Assignment	E_B (eV)	FWHM (eV)	At. %
Cu-MOF	Cu 2p$_{3/2}$ $_{Cu+}$	932.5	1.6	15.7
	Cu 2p$_{3/2}$ $_{Cu2+}$	934.7	2.1	84.3
	Satellite $_{Cu2+}$	939.2	4.0	-
	Satellite $_{Cu2+}$	943.7	3.5	-
Ag-Cu-MOF	Cu 2p$_{3/2}$ $_{Cu+}$	931.9	1.5	5.4
	Cu 2p$_{3/2}$ $_{Cu2+}$	934.3	2.5	94.6
	Satellite $_{Cu2+}$	939.2	4.0	-
	Satellite $_{Cu2+}$	943.4	3.3	-
Ca-Cu-MOF	Cu 2p$_{3/2}$ $_{Cu+}$	932.4	1.7	23.0
	Cu 2p$_{3/2}$ $_{Cu2+}$	934.6	2.2	77
	Satellite $_{Cu2+}$	938.8	4.0	-
	Satellite $_{Cu2+}$	943.6	3.5	-
Mn-Cu-MOF	Cu 2p$_{3/2}$ $_{Cu+}$	932.1	1.7	20.1
	Cu 2p$_{3/2}$ $_{Cu2+}$	934.4	2.5	79.9
	Satellite $_{Cu2+}$	938.9	4.0	-
	Satellite $_{Cu2+}$	943.6	3.5	-
Co-Cu-MOF	Cu 2p$_{3/2}$ $_{Cu+}$	932.5	1.7	23.2
	Cu 2p$_{3/2}$ $_{Cu2+}$	934.7	2.5	76.8
	Satellite $_{Cu2+}$	939.1	4.0	-

	**Satellite** $_{Cu2+}$	943.8	3.5	-
**Zn-Cu-MOF**	**Cu 2p**$_{3/2}$ $_{Cu+}$	932.3	1.7	27.0
	**Cu 2p**$_{3/2}$ $_{Cu2+}$	934.6	2.5	73.0
	**Satellite** $_{Cu2+}$	938.9	4.0	-
	**Satellite** $_{Cu2+}$	943.6	3.5	-

Table S3. Room temperature H_2S adsorption capacity comparison with the reported Cu-BTC.

Adsorbent	[H_2S] (ppm)	BTP (%)	q_e (mg g^{-1})
**MOF-199** [1]	500	100	77.1
**MOF-199** [2]	423	1	56.8
**MOF-199** [3]	10	100	69.0
**MOF-199** [4]	282	1	57.2
**CuBTC** [5]	1000	100	115.6
**CuBTC** [6]	500	80	27.1
**HKUST-1** [7]	10000	100	41.1
**HKUST-1** [8]	1000	10	92.0
**HKUST-1** [9]	99.6	5	23.8
**Ag-Cu-BTC** [10]	500	20	69.7
**Cu-BTC**<br>**Ag-Cu-BTC** [This study]	500	2	35.3<br>59.1

^{BTP} Breakthrough point; $^{N.A.}$ Not available

Table S4. Kinetic parameters for the IPD model.

MOFs	k_{ip} (mg g^{-1} min$^{-1/2}$)	C_i (mg g^{-1})	R^2
**Cu-BTC**	2.11	-2.71	0.99
**Ag-Cu-BTC**	1.46	-1.58	0.99
**Mn-Cu-BTC**	2.83	-0.29	0.99
**Co-Cu-BTC**	2.71	-0.52	0.99
**Ca-Cu-BTC**	2.69	-0.21	1.00
**Zn-Cu-BTC**	2.89	0.90	0.99

References

1. Gupta, N.K.; Bae, J.; Kim, K.S. From MOF-199 Microrods to CuO Nanoparticles for Room-Temperature Desulfurization: Regeneration and Repurposing Spent Adsorbents as Sustainable Approaches. *ACS Omega* **2021**, doi:10.1021/acsomega.1c03712.
2. Zhang, H.-Y.; Yang, C.; Geng, Q.; Fan, H.-L.; Wang, B.-J.; Wu, M.-M.; Tian, Z. Adsorption of Hydrogen Sulfide by Amine-Functionalized Metal Organic Framework (MOF-199): An Experimental and Simulation Study. *Appl. Surf. Sci.* **2019**, *497*, 143815, doi:10.1016/j.apsusc.2019.143815.
3. Lee, M.-H.; Vikrant, K.; Younis, S.A.; Szulejko, J.E.; Kim, K.-H. Chemisorption of Hydrogen Sulfide by Metal-Organic Frameworks and Covalent-Organic Polymers Based on Experimental/Theoretical Evaluation. *J. Cleaner Prod.* **2020**, *250*, 119486, doi:10.1016/j.jclepro.2019.119486.
4. Li, Y.; Wang, L.-J.; Fan, H.-L.; Shangguan, J.; Wang, H.; Mi, J. Removal of Sulfur Compounds by a Copper-Based Metal Organic Framework under Ambient Conditions. *Energy Fuels* **2015**, *29*, 298–304, doi:10.1021/ef501918f.
5. Peterson, G.W.; Britt, D.K.; Sun, D.T.; Mahle, J.J.; Browe, M.; Demasky, T.; Smith, S.; Jenkins, A.; Rossin, J.A. Multifunctional Purification and Sensing of Toxic Hydride Gases by CuBTC Metal–Organic Framework. *Ind. Eng. Chem. Res.* **2015**, *54*, 3626–3633, doi:10.1021/acs.iecr.5b00458.
6. Gupta, N.K.; Kim, S.; Bae, J.; Kim, K.S. Chemisorption of Hydrogen Sulfide over Copper-Based Metal–Organic Frameworks: Methanol and UV-Assisted Regeneration. *RSC Adv.* **2021**, *11*, 4890–4900, doi:10.1039/D0RA09017D.
7. Liu, J.; Wei, Y.; Li, P.; Zhao, Y.; Zou, R. Selective H_2S/CO_2 Separation by Metal–Organic Frameworks Based on Chemical-Physical Adsorption. *J. Phys. Chem. C* **2017**, *121*, 13249–13255, doi:10.1021/acs.jpcc.7b04465.
8. Petit, C.; Mendoza, B.; Bandosz, T.J. Hydrogen Sulfide Adsorption on MOFs and MOF/Graphite Oxide Composites. *ChemPhysChem* **2010**, *11*, 3678–3684, doi:10.1002/cphc.201000689.
9. Bhoria, N.; Basina, G.; Pokhrel, J.; Kumar Reddy, K.S.; Anastasiou, S.; Balasubramanian, V.V.; AlWahedi, Y.F.; Karanikolos, G.N. Functionalization Effects on HKUST-1 and HKUST-1/Graphene Oxide Hybrid Adsorbents for Hydrogen Sulfide Removal. *J. Hazard. Mater.* **2020**, *394*, 122565, doi:10.1016/j.jhazmat.2020.122565.
10. Gupta, N.K.; Bae, J.; Kim, K.S. Bimetallic Ag–Cu-Trimesate Metal–Organic Framework for Hydrogen Sulfide Removal. *New J. Chem.* **2021**, *45*, 22466–22477, doi:10.1039/D1NJ04601B.
